# Identification of Potential Protein Targets in Extracellular Vesicles Isolated from Chemotherapy-Treated Ovarian Cancer Cells

**DOI:** 10.3390/cimb45090469

**Published:** 2023-09-11

**Authors:** Chia-Yi Chan, Yi-Chun Ni, Hieu Duc Nguyen, Yung-Fu Wu, Kuen-Haur Lee

**Affiliations:** 1 Department of Nursing, Tri-Service General Hospital, National Defense Medical Center, Taipei 11490, Taiwan; 2 Graduate Institute of Cancer Biology and Drug Discovery, College of Medical Science and Technology, Taipei Medical University, Taipei 11031, Taiwan; 3 PhD Program for Cancer Molecular Biology and Drug Discovery, College of Medical Science and Technology, Taipei Medical University, Taipei 11031, Taiwan; 4 Department of Medical Research, Tri-Service General Hospital, National Defense Medical Center, Taipei 11490, Taiwan; 5 Cancer Center, Wanfang Hospital, Taipei Medical University, Taipei 11031, Taiwan; 6 TMU Research Center for Digestive Medicine, Taipei Medical University, Taipei 11031, Taiwan; 7 TMU Research Center of Cancer Translational Medicine, Taipei Medical University, Taipei 11031, Taiwan

**Keywords:** ovarian cancer, extracellular vesicle, chemoresistance, protein signature

## Abstract

Despite the ongoing clinical trials and the introduction of novel treatments over the past few decades, ovarian cancer remains one of the most fatal malignancies in women worldwide. Platinum- and paclitaxel-based chemotherapy is effective in treating the majority of patients with ovarian cancer. However, more than 70% of patients experience recurrence and eventually develop chemoresistance. To improve clinical outcomes in patients with ovarian cancer, novel technologies must be developed for identifying molecular alterations following drug-based treatment of ovarian cancer. Recently, extracellular vesicles (EVs) have gained prominence as the mediators of tumor progression. In this study, we used mass spectrometry to identify the changes in EV protein signatures due to different chemotherapeutic agents used for treating ovarian cancer. By examining these alterations, we identified the specific protein induction patterns of cisplatin alone, paclitaxel alone, and a combination of cisplatin and paclitaxel. Specifically, we found that drug sensitivity was correlated with the expression levels of ANXA5, CD81, and RAB5C in patients receiving cisplatin with paclitaxel. Our findings suggest that chemotherapy-induced changes in EV protein signatures are crucial for the progression of ovarian cancer.

## 1. Introduction

Global cancer statistics indicate that ovarian cancer is the third most fatal gynecological malignancy affecting women worldwide; a total of 313,959 new ovarian cancer cases and 207,252 deaths were reported globally in 2020 [1]. In Central and Eastern Europe, ovarian cancer has the highest incidence rate in the world (10.7/100,000) and a mortality rate of 5.6/100,000 [2]. There will be a worldwide increase of 55% in the incidence of ovarian cancer and an increase in ovarian cancer deaths of 67% based on population increase by 2035 [3]. Risk factors for ovarian cancer include infertility, endometriosis, obesity, age, and genetics (germline mutations in breast cancer susceptibility genes [*BRCA1/BRCA2*] and Lynch syndrome) [4]. The lifetime risk of BRCA1 caused by BRCA mutation is 40% to 60%, and the lifetime risk of BRCA2 is 11% to 27%. BRCA mutation can be detected in 14% to 18% of women with ovarian cancer, especially the high-grade serous ovarian cancer (HGSOC) subtype, which accounts for about 1% of the general population [5]. The symptoms of early-stage ovarian cancer are typically vague (such as indigestion and bloating), and because the ovary is small and located deep in the pelvic cavity, it is difficult to find the lesion which causes delayed referral for workup of malignancy [6]. Typically, no symptoms are observed during the early stages of ovarian cancer; moreover, clinical detection does not usually occur until later stages [7]. Beyond all the research being performed in ovarian cancer therapeutics, surgery is still a mainstay in the staging and treatment of ovarian cancer [8].

When the tumors are in advanced stages, which is the most usual situation, the standard of care for patients with ovarian cancer is cytoreductive surgery, followed by combination with chemotherapy [9]. Regimes have included cisplatin alone; a combination of doxorubicin, ifosfamide, dacarbazine, cyclophosphamide, and taxol; and various other combinations. Optimal cytoreduction followed by adjuvant platinum-based chemotherapy has been practiced based on case series and prospective trials [10]. Combination platinum chemotherapy in combination with paclitaxel, doxorubicin, ifosfamide, and other agents has been used with varying response rates. Approximately 80% of ovarian cancers are treated with cytoreductive surgery followed by adjuvant chemotherapy with carboplatin and paclitaxel or cisplatin and paclitaxel [11,12]. The drug mechanism of cisplatin is to prevent tumor growth by inhibiting the DNA synthesis of cancer cells; it is a non-specific cell cycle anti-tumor drug [13] and also the first gene complex to be approved by the US Food and Drug Administration (FDA) for the treatment of patients with OC. Response rates are good initially, but most patients treated with cisplatin eventually develop resistance through a variety of complex mechanisms, leading to treatment failure and increased mortality. Drug resistance may be caused by many reasons, including cell changes that occur before cisplatin binds to the cellular target as pre-target resistance, alterations of DNA–cisplatin adducts as on-target resistance, mutations or expression of downstream pathways that induce apoptosis changes as post-target drug resistance, and those not directly related to changes in cellular pathways and cisplatin-induced signals as off-target drug resistance [14]. Patient recurrence more than 6 months after front-line platinum-based therapy is considered platinum-sensitive, whereas platinum-resistant recurrence occurs after less than 6 months [15]. During the six months after the completion of major platinum-based chemotherapy, disease progression is usually closely related to platinum resistance. Due to its significant impact on patient survival time and quality, improving the response to platinum is an important challenge [16]. These chemotherapies are effective in treating the majority of patients with ovarian cancer. However, 70% of patients who receive this type of treatment relapse, and the recurring cancer is often resistant to standard platinum-based chemotherapy [17]. Because of its high recurrence and chemoresistance rates, 5-year survival rates in stage III and stage IV ovarian cancer are 42% and 26%, respectively [5]. Therefore, to improve the clinical outcomes of patients with ovarian cancer, new technologies must be developed for identifying the molecular alterations resulting from drug-based treatments of ovarian cancer.

During the past decade, the vesicles released by different cell types have been shown to be important mediators between the cells [18]. Long considered as inert debris or a hallmark of cell injury, extracellular vesicles (EVs) include apoptotic bodies, microvesicles, and exosomes [19]. Considering each EV subtype lacks specific markers, the International Society for Extracellular Vesicles has suggested the generic term “EVs” for the vesicles naturally released from the cells [20]. EVs are lipid–bilayer membrane-enclosed vesicles secreted by cells into the extracellular space with a diameter of 40–1000 nm [21]. However, the biogenesis process of EVs is very complex, and the mechanism underlying EVs formation and secretion remains poorly understood. EVs are intercellular transport carriers released under physiological and pathological conditions over long distances to recipient cells which can carry and deliver various molecules, such as nucleotides (DNA, RNA, mRNAs, miRNAs, etc.), proteins, lipids, metabolites, etc., which affect receptor cells [22,23]. EV contents can resist degradation under the pathological environment and cross the biological barrier with higher stability and bioavailability under the protection of the lipid bilayer structure of the membrane [24]. EVs encapsulate and convey information to surrounding cells or distant cells that are present in the surrounding extracellular environment through several mechanisms [25]. For example, some EVs can deliver their content through different types of endocytosis, such as clathrin-mediated endocytosis that is dependent or independent of receptors, macropinocytosis, and raft domain-mediated endocytosis [26]. In addition, EVs can also fuse with the membrane of the recipient cell to release their cargo intracellularly, either directly or through specific receptors [27]. Moreover, EVs may also release their contents into the extracellular space and activate a fast response in the neighboring cells [28]. Finally, the membrane surfaces of EVs can trigger signaling cascades through receptor/ligand interactions without internalization [29]. Thus, EVs have the potential to deliver complex information to multiple cells in their tissue environment, depending on both the cellular source and the stimulus that engendered their biogenesis [30].

Multiple studies have indicated that cancer cells release higher amounts of EVs compared to non-malignant cells, which makes the EV biogenesis machinery or components thereof attractive targets for anticancer therapy [31]. The ways by which tumor-derived EVs are involved in tumor growth are numerous and include both the uptake of EVs carrying oncogenic material (such as RNA or protein) by tumor cells and inhibiting the release from normal cells of EVs with tumor-suppressive cargo [32]. In addition, tumor-derived EVs with protumorigenic activity regulate cancer development by promoting cancer aggressiveness, invasiveness, angiogenesis, and drug resistance [33], suggesting the important effects of tumor-derived EVs on cancer development, progression, and therapy. EV-mediated therapy resistance can potentially act through distinct but not mutually exclusive mechanisms, including transfer of proteins and miRNA that promote therapy resistance and transfer of drug transporters, act as decoys for antibody-based therapeutics, and prevent antibodies from accessing their ligand target [34,35]. Therefore, identifying prognostic biomarkers capable of detecting drug response in patients with ovarian cancer may help improve their clinical outcomes.

In this study, we analyzed the changes in EV protein signatures due to different chemotherapeutic drugs used for the treatment of ovarian cancer. By examining these changes, we identified the specific protein induction patterns of cisplatin alone, paclitaxel alone, and cisplatin combined with paclitaxel. Drug sensitivity was found to be correlated with the expression levels of ANXA5, CD81, and RAB5C in patients receiving the combination of cisplatin with paclitaxel. Our findings suggest that chemotherapy-induced changes in EV protein signatures are crucial for the progression of ovarian cancer.

## 2. Materials and Methods

### 2.1. Cell Culture

The human ovarian cancer cell line ES2 was obtained from the Bioresource Collection and Research Center (Hsinchu, Taiwan). The cells were propagated in Roswell Park Memorial Institute 1640 medium (Life Technologies, Rockville, MD, USA) supplemented with 5% fetal bovine serum (Life Technologies, Rockville, MD, USA). Conditioned media containing ES2 cells treated with chemotherapeutic agents (10 µM for 24 h) were cultured in serum-free media for EV isolation.

### 2.2. EV Extraction and Size Determination

To isolate EVs from the ES2 cells, conditioned media containing ES2 cells treated with cisplatin alone, paclitaxel alone, and cisplatin combined with paclitaxel were collected. All media were centrifuged at 700× *g* to pelletize debris and cells; the supernatant was concentrated 1000 folds (by using centrifugal filter units; protein size cutoff: 100 kD) to a final volume of ≤500 μL. Subsequently, size exclusion chromatography with qEV columns (Izon Science, Christchurch, New Zealand) was performed as per the manufacturer’s instructions to separate the EVs from other supernatant constituents. Next, fractions containing EVs (fractions 1–3 after void volume) were pooled. The protein content was evaluated through a protein assay (Bio-Rad Laboratories, Hercules, CA, USA). Finally, to determine the size of the EVs, the nanoparticle tracking analysis was conducted using the qNANO instrument (Izon Science, Christchurch, New Zealand) as per the manufacturer’s instructions.

### 2.3. Proteomic Analysis of EVs

Complete proteomic profiling of EVs was performed through liquid chromatography (LC) with tandem mass spectrometry (MS/MS). Desalted peptides were subjected to LC-MS/MS by using an Orbitrap Elite hybrid ion trap/Orbitrap tandem mass spectrometer equipped with a 1D-LC (RP) Dionex UltiMate 3000 RSLCnano system (Tools Biotech, New Taipei City, Taiwan). Raw MS/MS spectra were analyzed using the Proteome Discoverer software (version 1.4; Thermo Fisher Scientific, Waltham, MA, USA). Then, for peptide identification, the MS/MS spectra were subjected to a search against the UniProt database (released on March 16, 2016; extracted for *Homo sapiens*; 20,199 sequences) by using the Mascot search engine (version 2.5, Matrix Science, London, UK). All proteins detectable by at least one unique peptide were deemed to be present in the sample (Table S1).

### 2.4. Gene Ontology and Functional Analysis

Specific upregulated and downregulated proteins in ovarian cancer cells treated with cisplatin combined with paclitaxel were subjected to a gene ontology (GO) analysis, which was performed using the WEB-based GEne SeT AnaLysis Toolkit (WebGestalt; http://www.webgestalt.org/option.php, accessed on 30 March 2021) [36]. Functional analysis of the aforementioned proteins in ovarian cancer cells treated with cisplatin combined with paclitaxel was conducted using the Enrichr [37] and ShinyGO [38] databases.

### 2.5. Cancer Treatment Response Gene Signature DataBase Analysis

The Cancer Treatment Response gene signature DataBase (http://ctrdb.ncpsb.org.cn/, accessed on 10 October 2022)—a unique tool for basic and clinical researchers to access, integrate, and reuse clinical transcriptome data pertaining to cancer drug response [39]—was used to determine the predictive values of ANXA5, CD81, and RAB5C for the chemotherapeutic sensitivity of ovarian cancer.

### 2.6. Statistical Analysis

For all data, significance was calculated using the one-sided Student *t*-test. A *p* value of <0.05 was considered to be statistically significant.

## 3. Results

### 3.1. Sizes of EVs Isolated from Drug-Treated ES2 Cells

**Figure 1:**
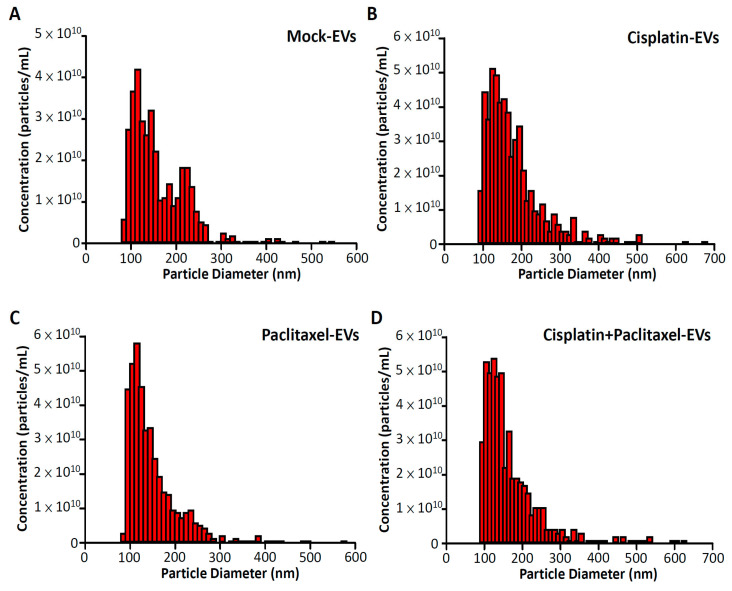
Particle size distribution and number of EVs isolated from ES2 cells treated with different chemotherapeutic agents. The figure shows representative histograms of the size distribution of EVs in mock ES2 cells (**A**), ES2 cells treated with cisplatin alone (**B**), ES2 cells treated with paclitaxel alone (**C**), and ES2 cells treated with both cisplatin and paclitaxel (**D**).

To determine the characteristics of EVs produced after the treatment of ovarian cancer cells with different chemotherapeutic agents, we examined the quantity and quality of these EVs. The nanoparticle tracking analysis revealed a multimodal distribution of particles ranging from 80 to 700 nm (mean value: approximately 180 nm, Figure 1). The number and particle size distribution were higher for the EVs isolated from ES2 cells treated with cisplatin alone than for those isolated from ES2 cells treated with paclitaxel alone and with both cisplatin and paclitaxel (Figure 1B–D).

### 3.2. Differential Expression of EV Proteins in ES2 Cells Treated with Various Chemotherapeutic Drugs

**Figure 2:**
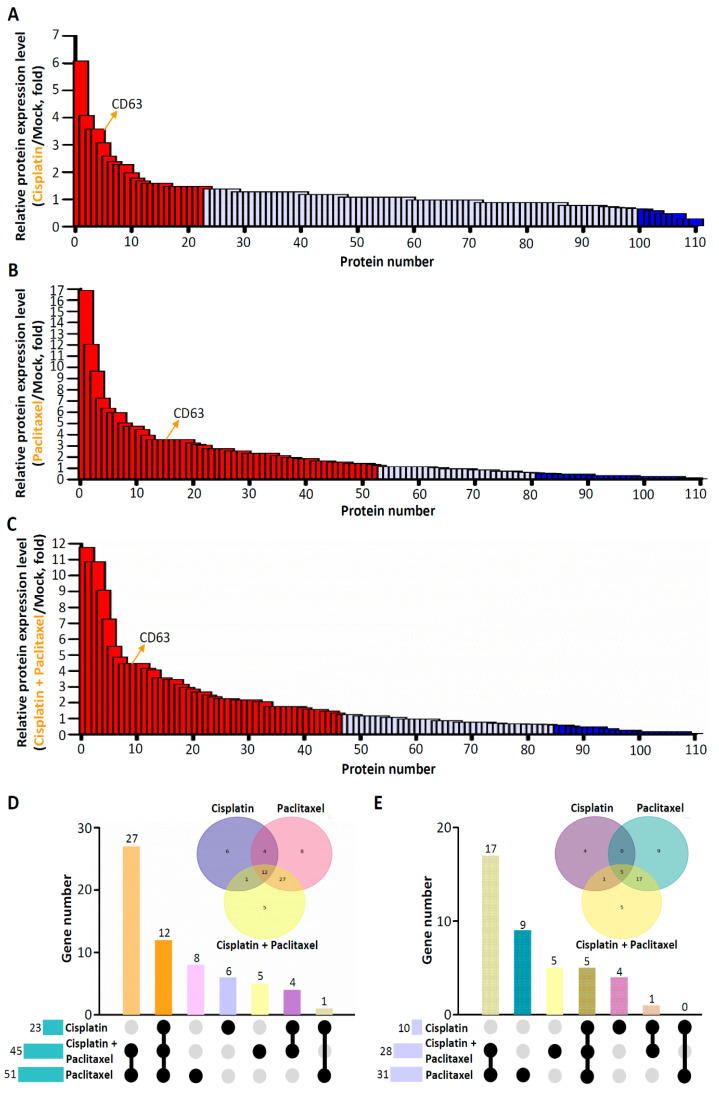
Protein expression levels in ES2 cells treated with different chemotherapeutic agents. The figure shows differentially expressed proteins in EVs isolated from ES2 cells treated with cisplatin alone (**A**), ES2 cells treated with paclitaxel alone (**B**), and ES2 cells treated with both cisplatin and paclitaxel (**C**) (unpaired Student’s *t* test, *p* < 0.05, with a fold-change cutoff of ≥1.5 for upregulation and downregulation). (**D**) Protein number refers to the number of upregulated proteins in each pair of ES2 cells treated with different chemotherapeutic agents. The Wayne diagram depicts the intersection of differentially expressed genes. (**E**) Protein number refers to the number of downregulated proteins in each pair of ES2 cells treated with different chemotherapeutic agents. The Wayne diagram shows the intersection of differentially expressed genes.

We used mass spectrometry to characterize the protein signatures of EVs isolated from ES2 cells treated with different chemotherapeutic agents. In accordance with the statistical analysis results pertaining to protein expression levels (unpaired Student’s *t*-test, *p* < 0.05, with a fold-change cutoff of ≥1.5 for upregulation and downregulation), we identified 23 instances of upregulated (red) and 10 instances of downregulated (blue) EV proteins from the comparison between cisplatin-treated and mock ES2 cells, 51 upregulated (red) and 31 downregulated (blue) EV proteins from the comparison between paclitaxel-treated and mock ES2 cells, and 45 upregulated (red) and 28 downregulated (blue) EV proteins from the comparison between cisplatin–paclitaxel-treated and mock ES2 cells (Table 1; Figure 2A–C). The mass spectrometry results revealed the expression of the general surface marker CD63 on the EVs isolated from ES2 cells treated with different chemotherapeutic agents (Figure 2A–C). To identify molecular alterations following the drug-based treatment of ES2 cells, we compared the posttreatment expression data of EV proteins among the different treatments. The results indicate that 12 proteins were upregulated in response to chemotherapy; of them, five specific proteins were upregulated in ES2 cells treated with both cisplatin and paclitaxel (Figure 2D; Table 1). Five proteins exhibited contrasting expression levels in response to chemotherapy; five specific proteins were downregulated in ES2 cells treated with both cisplatin and paclitaxel (Figure 2E; Table 2).

### 3.3. Results of GO and Functional Enrichment Analyses of Specific Deregulated Proteins in EVs Isolated from ES2 Cells Treated with Both Cisplatin and Paclitaxel

**Figure 3:**
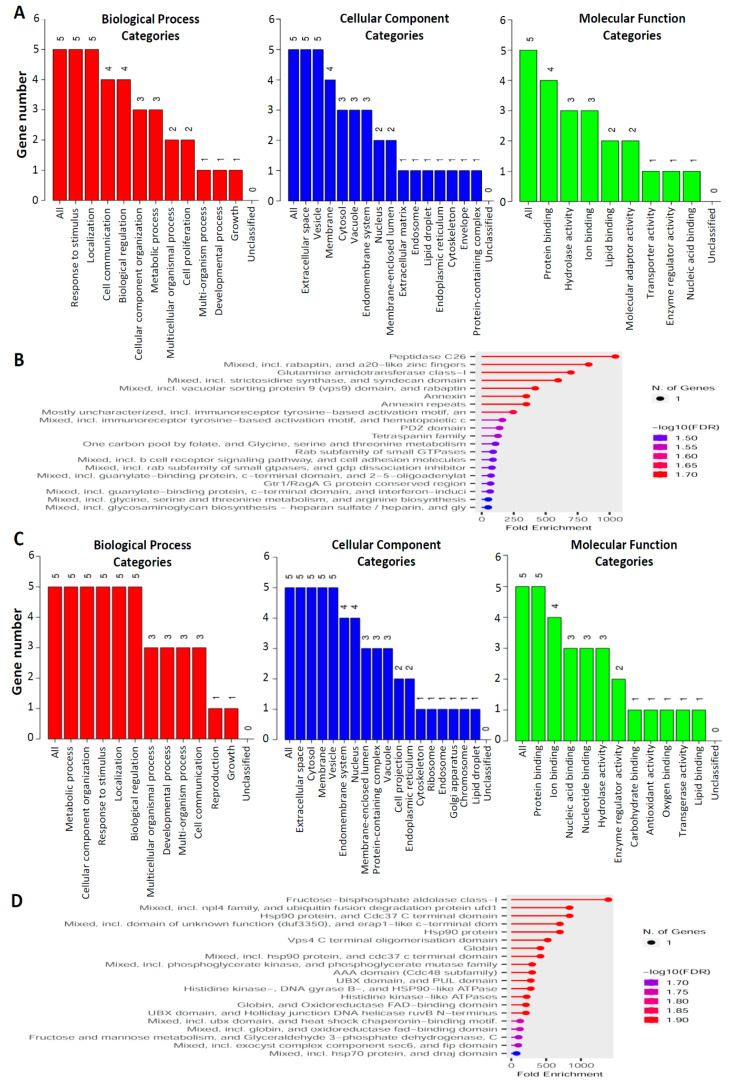
Results of GO and functional enrichment analyses of the top five upregulated and downregulated proteins in EVs isolated from ES2 cells treated with both cisplatin and paclitaxel. Results of the GO analysis of the top-five upregulated proteins (**A**) and top five downregulated proteins (**C**) Results of the pathway enrichment analysis of five upregulated proteins (**B**) and five downregulated proteins (**D**). The results are presented in the following three categories: biological process, cellular component, and molecular function. Each bar represents the number of proteins. GO, gene ontology.

Five specific proteins were upregulated in EVs isolated from ES2 cells treated with both cisplatin and paclitaxel; these proteins were further analyzed using WebGestalt [40] and ShinyGO [38]. For these proteins, the most prominent biological process–related GO terms were “response to stimulus” and “localization” (Figure 3A, left panel), the most prominent cellular component–related GO terms were “extracellular space” and “vesicle” (Figure 3A, middle panel), and the most prominent molecular function–related GO term was “protein binding” (Figure 3A, right panel). Figure 3B presents a chart graph depicting the relationships between these five specific upregulated proteins and enriched pathways. Five specific proteins were downregulated in ES2 cells treated with both cisplatin and paclitaxel. For these proteins, the most prominent biological process–related GO terms were “metabolic process”, “cellular component organization”, “response to stimulus”, “localization”, and “biological regulation” (Figure 3C, left panel); the most prominent cellular component–related GO terms were “extracellular space”, “cytosol”, “membrane”, and “vesicle” (Figure 3C, middle panel); and the most prominent molecular function–related GO term was “protein binding” (Figure 3C, right panel). Figure 3D depicts the relationships between these five specific downregulated proteins and enriched pathways.

### 3.4. Results of Drug Sensitivity and Response Analyses of Deregulated Proteins in EVs Isolated from ES2 Cells Treated with Both Cisplatin and Paclitaxel

**Figure 4:**
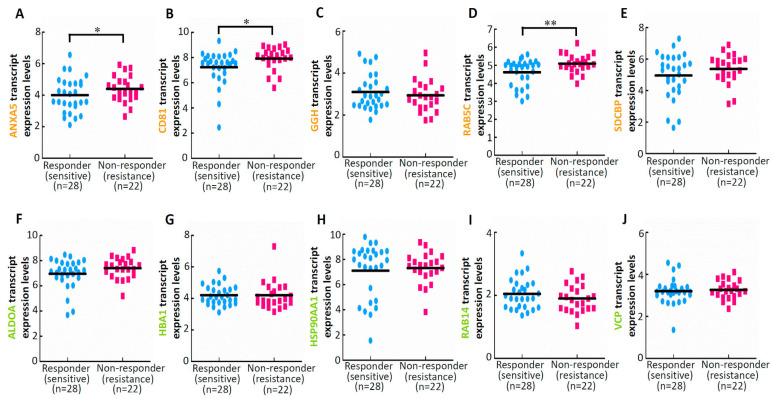
Results of drug sensitivity analysis of differentially expressed proteins in EVs isolated from cisplatin-treated ES2 cells. (**A**–**E**) Expression levels of five upregulated proteins (genes) in response to the combination of cisplatin and paclitaxel. (**F**–**J**) Expression levels of five downregulated proteins (genes) in response to the combination of cisplatin and paclitaxel. * *p* < 0.05 and ** *p* < 0.01.

We extracted data from the Cancer Treatment Response gene signature DataBase [41] and identified the correlations between the expression of deregulated proteins (genes) and drug sensitivity. Among the five specific upregulated proteins (genes) in EVs isolated from ES2 cells treated with both cisplatin and paclitaxel, ANXA5 (Figure 4A), CD81 (Figure 4B), and RAB5C (Figure 4D) were significantly upregulated in chemoresistant patients compared with their expression levels in chemosensitive patients (GSE30161). However, we observed no correlations between drug sensitivity and the expression levels of the five specific downregulated proteins (genes) in EVs isolated from ES2 cells treated with both cisplatin and paclitaxel (Figure 4F–J).

## 4. Discussion

Despite the ongoing clinical trials and the introduction of novel treatments over the past few decades, ovarian cancer remains one of the most fatal malignancies in women worldwide [42]. Currently, platinum- and taxane-based chemotherapy is regarded as the treatment of choice for most patients with ovarian cancer [43]. However, despite the initial high response rates, standard chemotherapeutic approaches are associated with recurrence in the majority of patients [44]. Therefore, the current limitations of chemotherapeutic treatment options necessitate the development of novel therapeutic strategies.

Recent studies have indicated that EVs display multiple roles in tumor progression [45]. EVs secreted by different kinds of cells are a kind of vesicles consisting of lipid bilayer membranes and play important roles in cell-to-cell communication [46]. EVs from mesenchymal stem cells could transfer angiogenesis-related microRNAs [47]. Metastatic organotropism is associated with EVs and the integrins of EVs could be used to predict tumor metastasis [48]. Until now, most studies have focused on EVs’ microRNA transfer in various cancers. It is known that the functions of EVs are not restricted to maintaining normal biological processes but also encompass drug resistance [45]. However, the mechanisms by which proteins in exosomes affect the phenotype of recipient cells due to complicated and variable biological processes and the mechanisms of chemoresistance are still elusive [49]. EVs mediate drug resistance through various mechanisms, including drug sequestration [50] and protein or RNA transfer [51,52,53]. The protein signatures of EVs isolated from drug-resistant tumors vary from those of EVs isolated from drug-sensitive tumors [54]. Therefore, alterations in specific EV proteins can be used as a prognostic and diagnostic biomarker of cancer. Accumulating research indicates that EVs are the important vesicles disseminating drug resistance. MicroRNAs in EVs, which could change various pathways related to chemotherapy resistance, have been reported in different cancers [55]. Previous studies have shown that EVs secreted by bone marrow stromal cells (BMSCs), cancer-associated fibroblasts (CAFs), and tumor cells promote chemotherapy resistance in human tumors [56,57,58]. For instance, some researchers have shown that transient receptor potential channel 5 (TrpC-5)-containing EVs in breast cancer and P-glycoprotein (P-gp)-containing microvesicles in ovarian cancer are responsible for chemotherapeutic resistance [59,60]. Glutathione S-transferase P1 (GSTP1), which is associated with detoxification and glutathione conjugation, has been reported in adriamycin-resistant breast cancer cells [61,62]. However, in the above studies, the functional proteins were selected by subjective conjecture instead of screening objectively. Thus, only some well-known proteins were identified, and novel and pivotal components in the EVs were not explored. Notably, the transmission of proteins by EVs is significant in regulating chemotherapy resistance.

A study by Zhao et al. found from patient samples that Midkine is a potential diagnostic marker in ovarian cancer for cisplatin/paclitaxel combination clinical therapy [63]. In our study, we examined the differential expression of EV proteins following the treatment of ovarian cancer cells with different chemotherapeutic agents by mass spectrometry analysis. We found that drug sensitivity was correlated with the expression levels of ANXA5, CD81, and RAB5C in patients receiving both cisplatin and paclitaxel. ANXA promotes resistance to several drugs, which indicates its importance in treatment resistance [64]. We found that the expression of ANXA5 was significantly upregulated in chemoresistant patients receiving platinum- and paclitaxel-based chemotherapy compared to chemosensitive patients receiving the same therapy. This finding is consistent with those of a study reporting an association between ANXA5 and drug resistance in ovarian cancer [15]. The mechanisms underlying ANXA-mediated drug resistance remain unclear. Recent research points out that the application of fusion protein combines ANXA5, an ovarian tumor- and tumor vasculature-targeting protein, with mutated cystathionine gamma-lyase (mCTH), an enzyme that converts selenomethionine (SeMet) into toxic methylselenol, which generates reactive oxygen species, leading to eventual tumor cell death [65]. Altogether, targeting ANXA5 may help eliminate drug resistance and improve treatment efficacy. However, most drug studies involving ANXA proteins are still in the laboratory stage, with very few clinical applications. Although the role of CD81 in the drug resistance of ovarian cancer remains unclear, studies have indicated a correlation between CD81 and drug resistance in patients with gastric cancer [66]. Hence, CD81 can be used as a therapeutic target to eliminate drug resistance and increase drug sensitivity. However, research on the clinical application of CD81 is still in its infancy. RAB5C is a guanosine triphosphatase that participates in endosomal membrane fusion reactions and can regulate endosome sorting [67]. This compound plays a role in tumorigenesis by promoting the migration of tumor cells [68]. Onodera et al. [69] reported that RAB5C promotes the invasion of breast cancer cells. However, the role of RAB5C in drug resistance remains unclear. Our results indicate a negative correlation between an elevated expression level of RAB5C and the sensitivity of patients with ovarian cancer to platinum- and paclitaxel-based chemotherapeutic regimens. Therefore, downregulating the expression of RAB5C may aid in the treatment of ovarian cancer.

## 5. Conclusions

Aberrant expression of ANXA5, CD81, and RAB5C affects the sensitivity of ovarian cancer cells to platinum- and paclitaxel-based chemotherapeutic agents. Therefore, ANXA5, CD81, and RAB5C may serve as therapeutic targets in drug-resistant ovarian cancer.

## Figures and Tables

**Table 1:** Upregulation of proteins in EVs isolated from ES2 cells treated with different chemotherapeutic agents.

Protein ID	Gene Name	Cisplatin	Paclitaxel	Cisplatin + Paclitaxel
P08133	ANXA6	ANXA6	—	—
P35613	BSG	BSG	—	—
Q01518	CAP1	CAP1	—	—
P68104	EEF1A1	EEF1A1	—	—
P06733	ENO1	ENO1	—	—
P07195	LDHB	LDHB	—	—
P60709	ACTB	—	ACTB	—
P68032	ACTC1	—	ACTC1	—
P04083	ANXA1	—	ANXA1	—
P06899	H2BC11	—	H2BC11	—
O60814	H2BC12	—	H2BC12	—
Q86YZ3	HRNR	—	HRNR	—
P11279	LAMP1	—	LAMP1	—
P02788	LTF	—	LTF	—
P08758	ANXA5	—	—	ANXA5
P60033	CD81	—	—	CD81
Q92820	GGH	—	—	GGH
P51148	RAB5C	—	—	RAB5C
O00560	SDCBP	—	—	SDCBP
P25311	AZGP1	AZGP1	AZGP1	AZGP1
P08962	CD63	CD63	CD63	CD63
P01040	CSTA	CSTA	CSTA	CSTA
P20930	FLG	FLG	FLG	FLG
P01859	IGHG2	IGHG2	IGHG2	IGHG2
P01834	IGKC	IGKC	IGKC	IGKC
P0DOY2	IGLC2	IGLC2	IGLC2	IGLC2
P13473	LAMP2	LAMP2	LAMP2	LAMP2
P61626	LYZ	LYZ	LYZ	LYZ
Q06830	PRDX1	PRDX1	PRDX1	PRDX1
P60900	PSMA6	PSMA6	PSMA6	PSMA6
P31151	S100A7	S100A7	S100A7	S100A7
P04075	ALDOA	ALDOA	ALDOA	—
P69905	HBA1	HBA1	HBA1	—
P00338	LDHA	LDHA	LDHA	—
P0CG48	UBC	UBC	UBC	—
P61204	ARF3	ARF3	—	ARF3
P02768	ALB	—	ALB	ALB
P07355	ANXA2	—	ANXA2	ANXA2
P05089	ARG1	—	ARG1	ARG1
P31944	CASP14	—	CASP14	CASP14
P04040	CAT	—	CAT	CAT
P07339	CTSD	—	CTSD	CTSD
P81605	DCD	—	DCD	DCD
Q08554	DSC1	—	DSC1	DSC1
Q02413	DSG1	—	DSG1	DSG1
P15924	DSP	—	DSP	DSP
Q01469	FABP5	—	FABP5	FABP5
Q5D862	FLG2	—	FLG2	FLG2
P04406	GAPDH	—	GAPDH	GAPDH
O75223	GGCT	—	GGCT	GGCT
Q16777	H2AC20	—	H2AC20	H2AC20
P14923	JUP	—	JUP	JUP
P31025	LCN1	—	LCN1	LCN1
P12273	PIP	—	PIP	PIP
P53801	PTTG1IP	—	PTTG1IP	PTTG1IP
P05109	S100A8	—	S100A8	S100A8
P06702	S100A9	—	S100A9	S100A9
O95969	SCGB1D2	—	SCGB1D2	SCGB1D2
Q96P63	SERPINB12	—	SERPINB12	SERPINB12
P29508	SERPINB3	—	SERPINB3	SERPINB3
P22735	TGM1	—	TGM1	TGM1
Q08188	TGM3	—	TGM3	TGM3
P10599	TXN	—	TXN	TXN

**Table 2:** Downregulation of proteins in EVs isolated from ES2 cells treated with different chemotherapeutic agents.

Protein ID	Gene Name	Cisplatin	Paclitaxel	Cisplatin + Paclitaxel
P04040	CAT	CAT	—	—
Q00610	CTSD	CTSD	—	—
P02788	LTF	LTF	—	—
P22735	TGM1	TGM1	—	—
P60033	CD81	—	CD81	—
Q99829	CPNE1	—	CPNE1	—
O75131	CPNE3	—	CPNE3	—
P0DMV8	HSPA1A	—	HSPA1A	—
P05556	ITGB1	—	ITGB1	—
P00558	PGK1	—	PGK1	—
P51148	RAB5C	—	RAB5C	—
O00560	SDCBP	—	SDCBP	—
P02786	TFRC	—	TFRC	—
P04075	ALDOA	—	—	ALDOA
P69905	HBA1	—	—	HBA1
P07900	HSP90AA1	—	—	HSP90AA1
P61106	RAB14	—	—	RAB14
P55072	VCP	—	—	VCP
P54709	ATP1B3	ATP1B3	ATP1B3	ATP1B3
Q00610	CLTC	CLTC	CLTC	CLTC
P60842	EIF4A1	EIF4A1	EIF4A1	EIF4A1
Q08380	LGALS3BP	LGALS3BP	LGALS3BP	LGALS3BP
Q9NZM1	MYOF	MYOF	MYOF	MYOF
Q86YZ3	HRNR	HRNR	—	HRNR
O43707	ACTN4	—	ACTN4	ACTN4
P05023	ATP1A1	—	ATP1A1	ATP1A1
P35613	BSG	—	BSG	BSG
P62879	GNB2	—	GNB2	GNB2
P04439	HLA-A	—	HLA-A	HLA-A
P26006	ITGA3	—	ITGA3	ITGA3
P26038	MME	—	MME	MME
Q9NZM1	MSN	—	MSN	MSN
O75340	PDCD6	—	PDCD6	PDCD6
Q8WUM4	PDCD6IP	—	PDCD6IP	PDCD6IP
P62937	PPIA	—	PPIA	PPIA
P26022	PTX3	—	PTX3	PTX3
P61586	RHOA	—	RHOA	RHOA
P27105	STOM	—	STOM	STOM
P68363	TUBA1B	—	TUBA1B	TUBA1B
P62258	YWHAE	—	YWHAE	YWHAE
P63104	YWHAZ	—	YWHAZ	YWHAZ

## Data Availability

MDPI Research Data Policies.

## Supplementary Materials

The following supporting information can be downloaded at https://www.mdpi.com/article/10.3390/cimb45090469/s1. Table S1: Results of the mass spectrometry of EV proteins in ovarian cancer.
